# Photonic and electrochemical biosensors for near-patient tests–a critical comparison

**DOI:** 10.1364/OPTICA.530068

**Published:** 2024-10-04

**Authors:** Thomas F. Krauss, Lisa Miller, Christoph Wälti, Steven Johnson

**Affiliations:** 1 School of Physics, Engineering and Technology and York Biomedical Research Institute, University of York, York, UK; 2 School of Electronic and Electrical Engineering, University of Leeds, Leeds, UK

## Abstract

Research into diagnostic biosensors is a vibrant field that combines scientific challenge with translational opportunities; innovation in healthcare is of great societal interest and is an essential element of future healthcare provision. Photonic and electrochemical biosensors are the dominant modalities, both scientifically and commercially, yet the two scientific communities largely remain separated and siloed. It seems astute to better understand what the two fields can learn from one another so as to progress the key scientific, translational, and commercial challenges. Here, we provide an analysis of the fundamental operational characteristics of photonic and electrochemical biosensors using a classification based on energy transfer; in photonics, this separates refractive index sensors from fluorescence and vibrational spectroscopy, while in electrochemistry, it distinguishes Faradaic from non-Faradaic processes. This classification allows us to understand some of the key performance characteristics, such as the susceptibility to fouling and dependence on the clinical matrix that is being analyzed. We discuss the use of labels and the ultimate performance limits, and some of the unique advantages of photonics, such as multicolor operation and fingerprinting, and critically evaluate the requirements for translation of these technologies for clinical use. We trust that this critical review will inform future research in biosensors and support both scientific and commercial developments.

## 1. INTRODUCTION

Biosensors are a vibrant area of research that offer opportunities for academic exploration combined with the challenge and satisfaction of contributing to real-world problems; they ideally combine intellectual challenge with societal need. Arguably, the field started in 1962 with the first biosensor invented by Clark and Lyons who measured glucose in biological samples via the electrochemical detection of oxygen using an electrode coated with a layer of the glucose oxidase enzyme [1]. This work led to the ubiquitous glucose sensor that is used widely today for the management of diabetes. Another early biosensor modality is based on the phenomenon of surface plasmon resonance, which was first presented in the early 1980’s [2]. This technology was successfully commercialized resulting in the well-known Biacore instrument. These historical developments are reflected in the current market share, which is dominated by electrochemical sensors (72% market share worldwide), followed by optical (14%) and other modalities (14%) [3]. In terms of the research landscape, we note a similar picture with electrochemical and optical biosensors remaining the two dominant sensing modalities. A search for “electrochemical AND biosensor” yields approx. 9000 returns for the last 5 years on Web of Science, while “(optical OR photonic) AND biosensor” yields approx. 4000 returns. Relevant review papers produce a similar picture, e.g., [4] lists biosensors as electrochemical, optical, microgravimetric, magnetic, and thermal detection, in that order. This poses the question as to why these two modalities are so dominant, and why electrochemical sensors attract more attention. Is there a fundamental difference that makes electrochemical sensors more attractive than photonic counterparts? Superficially, the two modalities appear to be very distinct, but are there commonalities that can be exploited? Given the readership of Optica, is there something that the optics and photonics community can learn from electrochemistry?

Many review papers have been written on various photonic biosensor modalities, but it appears that photonics researchers rarely ask the question as to whether a non-photonic modality might be superior for a particular application, thereby missing an opportunity to learn from other fields. Moreover, many review papers mainly serve to provide a summary of the activities in a particular research field (or from the senior author’s research group), highlighting the many achievements that have been reported, while omitting the related limitations. While reporting achievements is clearly necessary, we suggest that highlighting trade-offs and disadvantages is as important for the reader to make an informed choice about where to direct their research effort. Therefore, the aim of this paper is to identify the fundamental differences, as well as the similarities, between photonic and electrochemical sensors and to identify some unique features.

## 2. CLASSIFICATION

We first look at the fundamental mechanisms that determine the operation of photonic and electrochemical sensors. We focus on sensors that interrogate surface-bound target molecules, also known as “surface affinity” biosensors, because they confer high sensitivity and high specificity, as we will detail later. Surface affinity biosensors employ a surface-immobilized binder molecule, such as an antibody or a DNA aptamer, that specifically binds to and captures a target molecule, localizing it close to the sensor surface. We will use the generic term “binder molecule” throughout, but we recognize that such molecules may also be referred to as bioreceptor, probe molecule, or capture agent. Using a binder molecule in conjunction with a surface affinity biosensor confers high performance and flexibility, because such a system can target a wide range of diseases simply by immobilizing a different binder molecule on the surface. The liquid containing the target molecule is here referred to as the “sample” while one tends to use “matrix” in the context of complex liquid samples such as clinical samples. We note that other publications also use the term “analyte” for the liquid carrier.

**Fig. 1.**
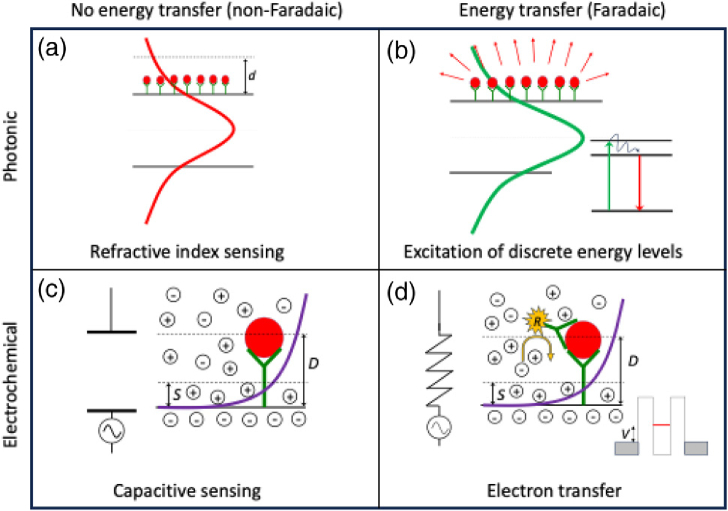
High-level classification of photonic and electrochemical sensing modalities, using energy transfer as the classifier. In (a) photonic refractive index sensing, the optical mode experiences the phase change imposed by the bound molecules, but the mode is not absorbed (no energy transfer). The phase change is then translated into a measurable output using resonant effects [5,6] or interferences [7–9]. In (b) fluorescence or vibrational spectroscopy, the molecular layer partially absorbs the mode, which is either detected directly, via a frequency shift (e.g., Raman) or via energy transfer to a fluorescent state, indicated here by the Jablonski diagram in the inset. In (c) non-Faradaic electrochemical detection, the capacitance of the electrochemical double layer D is modified by the presence of the bound antigen, and this change in capacitance is detected [10]. In (d) Faradic detection, the double layer is considered as a shunt resistor and the presence of a redox-active molecule gives rise to charge transfer upon application of a voltage, which is measured as a current. The redox active molecule is either immobilized directly to the electrode surface, or added to the electrolyte as a freely diffusing redox probe [11]. Energetically, Faradic processes are comparable to a resonant tunnelling diode, where electron tunnelling through a discrete state is achieved by applying a voltage. Approximate scales for the antigen-antibody layer are 5–15 nm, the decay length d of the optical mode is typ. 50–100 nm, and the Debye length D for typical electrolytes is in the range of 5–10 nm. Clearly, the schematic is a simplification, and combinations exist, e.g., between Faradaic and non-Faradaic methods, but this depiction allows us to draw out the fundamental properties.

We begin with a high-level classification of the sensing modalities. Both photonics and electrochemistry offer two distinct modes of operation, depending on whether energy is exchanged between the transducer and the target molecule. In photonics, this separates refractive index sensing from fluorescence and vibrational spectroscopy. In electrochemistry, it differentiates between Faradaic and non-Faradaic processes, i.e., whether charge is transferred to/from an electrode (as is the case in a Faradic process) or not (a non-Faradic process). In a Faradic process, the transfer of charge is typically associated with a chemical reduction or oxidation reaction, while non-Faradaic methods quantify changes in capacitance that occur when the target biomarker binds to the surface. Figure 1 illustrates this high-level classification.

An electrochemical double layer forms when the surface of an electrode is brought into contact with an electrolyte such as a buffer solution, urine, or serum. This ordered electrical interface is similar to the depletion layer between a conductor and a doped semiconductor. As in semiconductor junctions, the thickness of the depletion layer at the electrode-electrolyte interface is inversely proportional to the charge density or ionic strength. Since the charge density in a typical electrolyte is high, the depletion layer is thin, i.e., typically <10nm for an ionic electrolyte of concentration >1mM . In electrochemistry, this depletion layer is termed the “electrochemical double layer”. It is a “double” layer because it is formed by the combination of hydrated ions adhered to the electrode surface (“Stern layer”), S , and the diffusive layer limited by the Debye length, D .

The difference in length scale, given by the difference between the optical decay length, d , of the optical mode and the electrochemical Debye length, D , highlights an essential difference between the two modalities. The electrochemical potential overlaps more closely with the molecular layer than the optical field, so is in principle more sensitive to changes that occur at the surface. This close proximity also means, however, that electrochemistry is more susceptible to surface variability, non-specific binding to the sensor surface, and the properties of the sample, which we will explore further below.

### A. Example of Practical Sensors

**Fig. 2.**
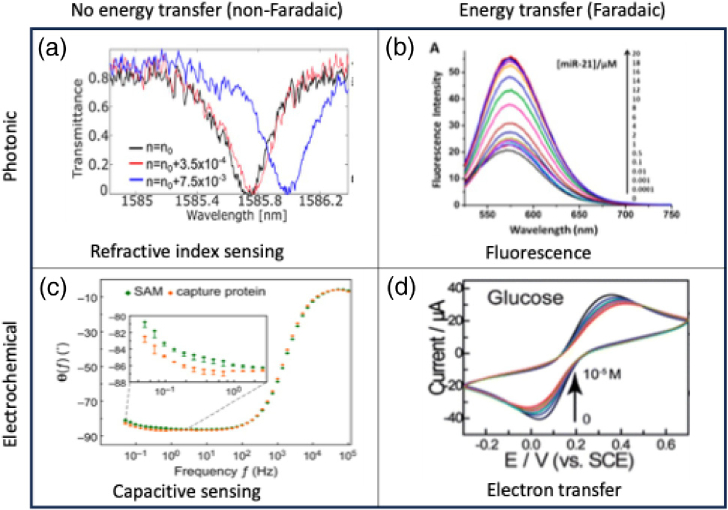
Examples of sensor readout for the classification as in Fig. 1. (a) Wavelength scan of a microring resonator for different refractive indices, reprinted from [12] following the terms of Creative Commons CC-BY4.0 license. (b) Fluorescence spectrum readout as a function of the concentration of fluorescently labelled micro-RNA, reprinted with permission from [13] copyright 2017, American Chemical Society. (c) Non-Faradic, impedance spectroscopy (here showing the shift in phase as a function of frequency) of an electrode with and without capture proteins, reprinted from [10] following the terms of Creative Commons CC-BY4.0 license. (d) Cyclic voltammogram (voltage scan) of a glucose sensor for varying concentrations of glucose, reprinted from [11] following the terms of Creative Commons 3.0 license.

Following the same classification as in Fig. 1, we give examples of the different types of photonic and electrochemical sensors in Fig. 2.

In the case of the microring resonator as in Fig. 2(a), the spectrum of the resonance is tracked as the refractive index of the cladding changes. The difference in center wavelength is then mapped onto the refractive index or biomarker concentration using suitable calibration curves. The figure nicely illustrates how the detection limit is determined by the readout noise. The fluorescence readout [Fig. 2(b)] measures the signal strength as a function of biomarker concentration, i.e., it is a direct intensity measurement. Note the presence of a background signal, i.e., this particular example features a fluorescence signal even in the absence of the target. Non-Faradaic electrochemical impedance spectroscopy (EIS) [Fig. 2(c)] scans the frequency of an applied ac voltage and measures both the amplitude and phase of the corresponding current to determine the impedance of the electrochemical double layer. In Fig. 2(c), we show the change in the phase of the impedance, which is more sensitive to changes in the electrochemical double layer, particularly at low frequencies as shown here. Specifically, the inset reports the difference in phase between an electrode surface prepared with a self-assembled monolayer (SAM) and an electrode in which the SAM is subsequently functionalized with a layer of binder molecules/capture proteins. The cyclic voltammogram in Fig. 2(d) shows how the redox current changes as a function of glucose concentration. As in Fig. 2(b), there is a background signal even in the absence of glucose and this background current, associated with charging of the electrochemical double layer capacitance, is a function of voltage sweep rate.

### B. Sensitivity, Specificity, and Non-specific Binding

The term “sensitivity” can have multiple meanings. In diagnostics, it refers to the probability of a diagnostic test to return a true positive result, e.g., 98% sensitivity means that the test can correctly detect the presence of a biomarker at the clinically relevant level with 98% certainty. In the physical sciences, “sensitivity” typically refers to the gradient of the response curve, e.g., photonics researchers discussing resonant sensors refer to sensitivity as the change of resonance wavelength (in nm) versus the change of refractive index (in refractive index units, RIU), so sensitivity S in this context is reported in units of nm/RIU. Here, we shall adopt the physical sciences definition and use the term to refer to the gradient of the transducer’s response. In addition, we note that the limit of detection (LOD) is typically defined as three times the noise level, which is used to describe the minimum detectable measurand and is expressed as a physical unit (current, voltage, refractive index units, such as $10^{-5}\;RIU$ ) or the minimum detectable concentration of a target molecule, expressed as a concentration (weight/volume or molar, such as 20 picomolar). As an aside, the conversion between molar and weight/volume units is referenced to a 1 kDa molecule, i.e., a 1 kDa molecule of 1 ng/ml is present at a 1 nM concentration; a 20 kDa molecule at 1 ng/ml would be present at 50 pM concentration.

The definition of sensitivity as a gradient is useful for describing the physical modality, but it fails to fully account for the response of a complete biosensor. A good example is the focus of many photonics papers on the figure of merit defined as the product of Q-factor and sensitivity [5], which, as an aside, we have recently shown to be only partially correct [12]. Critically, the performance of a photonic biosensor is not only dependent on the figure of merit of the photonic system, but also on the surface functionalization and the properties of the binder molecule. As a case in point, we recently compared the reported limits of detection of comparable photonic sensors, such as microring resonators and Mach-Zehnder interferometers [14] and, despite similar figures of merit in photonic performance, the diagnostic performance varied by several orders of magnitude, likely related to differences in approaches used to functionalize the sensor surfaces.

The binding between an antibody and its target antigen is an extremely specific process; this specificity has evolved over millions of years to confer immunity to a host through the specific targeting of proteins associated with a pathogen while avoiding initiation of an immune response by the host’s own proteins. Therefore, in a surface affinity biosensor, it is the antibody that confers specificity to the sensor, i.e., the ability to bind a specific biomarker and only this biomarker. However, antibodies have evolved to operate in solution. Once immobilized on a surface, interactions between the surface and the antibody and/or between adjacent antibodies tend to lead to a reduction in specificity and affinity. In addition, the surface restricts the free diffusion of target biomarkers towards their binding site. As a result, the measured binding affinity tends to be affected by the immobilization of the binder molecule to the surface [15,16]. In addition, depending on the materials that constitute the sensor and the chemistries used to immobilize the binder molecule, the surface itself can non-specifically bind a wide range of biomolecules present in a clinical matrix, further reducing the diagnostic specificity.

**Fig. 3.**
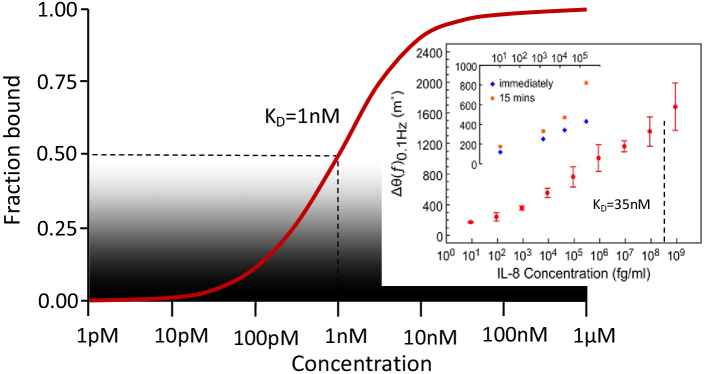
Binding curve of a typical binder molecule, which is specified via the dissociation constant, $K_{D}$ . $K_{D}$ describes the concentration of the target at which half of the binding sites are bound, in this example one nanomolar. The limit of detection of the sensor for a particular analyte is then determined by the $K_{D}$ of the surface immobilized antibody, the physical transduction mechanism, and the signal-to-noise ratio of the sensor. Please note that the value of $K_{D}$ varies widely as a function of the binder molecule and approaches used to attach the binders to the sensor surface, as well as on experimental parameters such as temperature and pH. Moreover, the readout is not necessarily linear with the fraction of molecules bound, e.g., it may be nonlinear and over-emphasize low concentrations to improve the limit of detection. The inset (adapted from [10], following the terms of Creative Commons CC-BY4.0 license) highlights the fact of how far the lower end of the concentration scale can be explored with a suitable technique, here non-Faradaic EIS. The biomarker is IL-8, which is a small protein associated with inflammatory diseases. The $K_{D}$ is 35 nM (indicated by the dotted line), or 300 ng/ml ( $3\times 10^{8}\;fg/ml$ ), which is near the maximum concentration shown in the inset.

The sensitivity and LOD are thus determined by a combination of the layer of surface-immobilized binder molecules and the performance of the physical sensor. This combination is illustrated by the Langmuir adsorption model shown schematically in Fig. 3. Here, the dissociation constant $K_{D}$ quantifies the affinity of the binder molecule. It is defined as the concentration at which half of the binder molecules are bound to their associated target molecule. $K_{D}$ sets the bounds within which the sensor can operate, and the sensitivity and signal-to-noise ratio of the sensor then determine the range of biomarker concentration over which the sensor usefully operates. This point is illustrated by the well-known Covid lateral flow tests, which are antibody-based and provide high specificity, but the trade-off for their simplicity is a relatively high LOD, which leads to a high fraction of false negative readouts. The same holds for the glucose sensor, where oxidation of glucose by the immobilized glucose oxidase enzyme is highly specific, but the limit of detection is not particularly low. In this case, there is no issue, because clinically relevant glucose levels are high, so high diagnostic performance can be achieved despite a high limit of detection.

## 3. PERFORMANCE AND FUNCTIONALITY

### A. Surface Quality

Figure 1 highlights the major difference between our two modalities with respect to surface interactions. In photonics, the optical field extends well beyond the surface and the interaction between the mode and the surface-bound molecules is given by the overlap between the two. A photonic sensor therefore mainly responds to changes in total bound mass, e.g., changes caused by molecular binding, whereas conformational changes, where the molecule changes shape but not mass, are more difficult to detect. In contrast, the surface potential of the electrochemical double layer extends to a similar distance as the immobilized molecular layer. This confers high sensitivity to desired binding events as well as conformational changes, but also to the surface quality. A good example for this observation is non-Faradaic EIS, where binding events are detected by changes in the impedance. This approach can be extremely sensitive [10], but it relies on the assembly of a uniform, high-impedance layer at the electrode interface, which in turn requires a high-quality electrode surface (in terms of the material purity, surface cleanliness and surface roughness). Moreover, for commercial applications, the layer needs to be made with high consistency and reproducibility, which is extremely challenging to achieve at scale. As a result, we are not aware of any high-performance, non-Faradaic impedance biosensors that are in clinical use.

In contrast, while Faradaic electrochemical sensors require surfaces that facilitate electron transfer between an electrode and the redox label, the requirement on surface quality is lower; this lower requirement enables the use of mass fabrication approaches such as screen-printed electrodes. Therefore, most electrochemical sensors are of the Faradaic type. The downside of Faradaic measurements is the requirement for the application of an external voltage, which always generates a background current, which limits the minimum signal that can be detected reliably and therefore the LOD.

In the case of the canonical Faradaic sensor, i.e., the glucose sensor, the electrochemically active molecule is the glucose oxidase enzyme, which is either directly bound to the surface, or in more recent implementations, is connected electrically to the surface via a redox shuttle, and well inside the Debye length, which makes the setup very robust and less dependent on surface quality. In contrast, most other targets of interest, such as protein biomarkers, are often not electrochemically active, so Faradic sensing requires the addition of an electrochemically active label. This requirement adds complexity to the operation of the sensor and it makes the readout dependent on the position of the label with respect to the electrochemical potential.

### B. Environmental Interferences and Fouling

So far, we have only considered sensing in a controlled sample, typically a laboratory buffer such as phosphate buffered saline (PBS), which is spiked with the biomarker of interest and in which the aqueous matrix is assumed to have little or no impact on the sensor performance. Reporting extremely low limits of detection in ideal analytes and in highly controlled environments is clearly an important scientific achievement, but it may be less relevant for future applications focused on clinical diagnostics. For example, Kabashin *et al.* have demonstrated an interferometric sensor based on surface plasmons that can detect changes in the refractive index as low as 1e-8 refractive index units [17,18]. This impressive result was achieved by using a gas mixture to control the refractive index. The same measurement, if it had been conducted in a real matrix, would have been screened by thermal and density fluctuations, so a very low physical limit of detection does not automatically translate into high clinical performance.

In addition to environmental fluctuations, a key reason why many surface affinity sensors are not limited by the fundamental performance of the transducer is the non-specific binding or “fouling” of the sensor surface. Hence, when targeting real disease and real healthcare applications, the sensor performance should ideally be assessed in real matrices, such as urine, serum, plasma, or blood. We note that both photonic and electrochemical sensors are susceptible to fouling, and the impact of fouling depends on the specific configuration and surface chemistry. As an example, we recently demonstrated protein biomarker detection limits in the pg/ml range in PBS, using a polydopamine-based surface functionalization protocol. When the same sensor and surface chemistry were used with spiked human serum, the limit of detection worsened to low ng/ml concentrations [14].

Nonspecific binding to a biosensor surface can lead to false positives or false negatives, depending on the format or the assay. In label-free sensing, the nonspecific binding of components other than the target biomarker can lead to a false positive reading. Conversely, with a labelling approach, nonspecific binding may prevent the secondary antibody from binding and will produce a false negative result.

The most obvious mitigation strategy against fouling is to use a control or reference channel and to functionalize it with an isotype antibody, i.e., to use an antibody of similar type as the one used to bind the target of interest, but without active binding sites. The assumption is that fouling will impact both the measurement and control channels equally such that the reference can be subtracted from the signal, thus accounting for fouling. This works reasonably well and was, e.g., used in [14], but has its limitations, for example, fouling can also interfere with the binding of the target to the antibody.

**Fig. 4.**
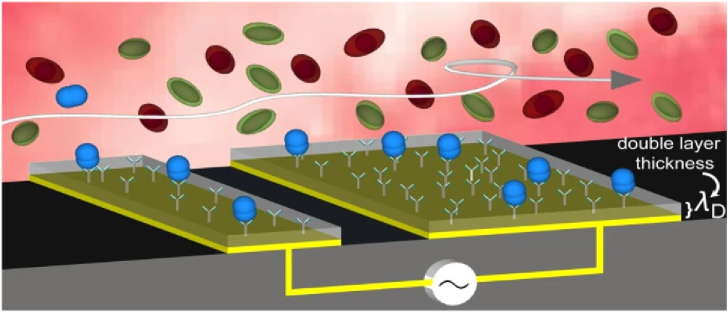
Schematic representation of electrically induced nanoshearing: the shear forces occur within nanometers ( $\lambda_{D}$ = double layer thickness) of the electrode surface, causing fluid flow that increases the number of high-affinity target-antibody collisions (blue) while shearing away the weaker nonspecifically bound molecules (green and red). Reprinted from [19], following the terms of Creative Commons CC-BY4.0 license.

Other mitigation methods broadly fall into two main approaches: a) sample processing or b) modifications to the sensor surface. Sample processing can be achieved by introducing additives such as Tween 20, a common surfactant, that will reduce nonspecific binding. The processing can also occur after the sample has been exposed to the sensor; washing buffers can be an effective method of removing the non-specifically bound components, which often have lower affinity for the sensing layer than that of the antibody-antigen interaction. Vaidyanathan *et al.* [19] were able to use this difference in binding affinities to both increase antigen capture and to displace weakly bound foulants by applying nanoshearing to their surface, using alternating current electrohydroynamics (Fig. 4). They report that such flow shearing results in a 1000-fold enhancement in biomarker detection in serum compared to standard flow. The method can be implemented as a lab-on-a-chip platform and both photonic and electrochemical biosensors can benefit.

**Fig. 5.**
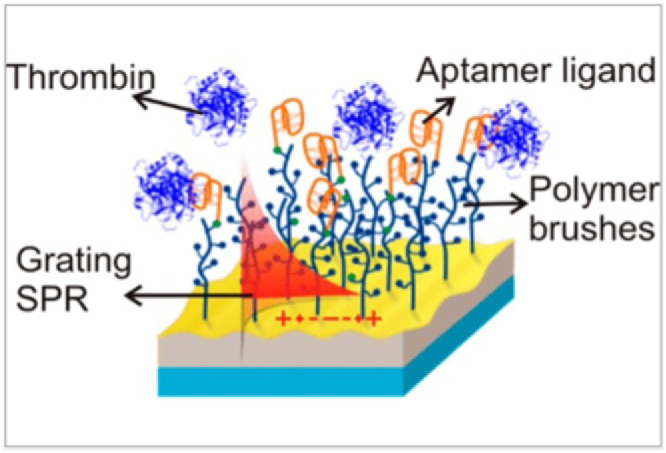
Schematic of a sensor chip surface decorated with nonfouling polymer brushes and aptamers for the detection of thrombin (reprinted from [20], following the terms of Creative Commons CC-BY4.0 license).

Immobilizing antifouling agents on the sensor surface is the more common approach to minimizing the non-specific binding of proteins to the sensor surface. Polyethylene glycol (PEG) molecules are often used because of their high hydrophilicity and the fact that PEG molecules easily form a hydration layer on the surface that inhibits protein adsorption. A more recent development is “polymer brushes”; polymer brushes protect the surface from foulants with minimal effect on the affinity of the binders as shown conceptually in Fig. 5. For example, Kotlarek *et al.* [20] evaluated fouling of a polymer-brush-modified optical biosensor for thrombin in human blood plasma. They reported no measurable fouling of the pure polymer brush (PB) surface in 100% plasma by SPR, whereas the more conventional PEG-coated surface led to significant fouling. Naturally, even a perfect antifouling surface is subject to non-specific binding when modified to include a binder molecule, because other molecules may non-specifically attach to the binder molecule. It is thus important to note that the choice of binder molecule can play a significant role in the antifouling properties, with larger binder molecules such as antibodies typically offering a higher risk of fouling.

Despite the plethora of methods to mitigate fouling that have been developed, we note that each method needs to be optimized for its specific matrix and binder molecule. This lack of universality makes it difficult to define a standard for measuring the antifouling abilities of a given strategy and to compare different antifouling approaches to identify the most appropriate. It also presents a barrier to commercialization; see Section 4.E.

As an aside, the measured limit of detection also depends on the fluidic system used to deliver a sample to the sensor surface. Biomarkers can readily physiosorb to flow cells and fluidic tubing, to the effect that they may not reach the sensor or at least at a reduced concentration; please see [21] for more detail in the context of the widely used PDMS microfluidics. Therefore, the detection limit of the sensor itself may actually be better than what is reported.

### C. Direct Matrix Dependence

An additional issue associated with the choice of measurement matrix is the susceptibility to ionic shielding, which mainly affects electrochemical modalities and means that sensors respond to changes in concentration and pH of the matrix. These changes directly impact on the Debye length [Figs. 1(c) and 1(d)] because of the dependence on matrix conductivity and the corresponding electrochemical response. In detail, a non-Faradaic sensor operates by applying an AC voltage and measuring the resulting current to determine the effective impedance of the surface, which may change either because of changes to the potential distribution, or because of binding events. Therefore, a change in matrix conductivity directly impacts the readout, and cannot be distinguished easily from changes due to binding. As a result, clinical samples from different patients can produce a different readout, even if the biomarker concentration is the same. In photonics, concentration and pH also affect the refractive index, but have a lower impact on the response to a binding event because the overlap of the mode with the molecular layer is not affected; concentration changes produce a step-change in readout response, but do not affect the binding curve. For example, we have demonstrated photonic detection of similar performance (LOD in the low pg/ml range) for PBS and urine [22], which suggests that urine is a less interfering matrix than serum or plasma.

Naturally, changes in pH also change the binding affinity of most binder molecules that have evolved or have been engineered to operate at physiological pH (pH 7.4), so their binding affinity will be impacted irrespective of the sensor modality.

In conclusion, fouling and the matrix dependence are some of the biggest issues for both modalities, although arguably more so for electrochemical sensors due to the impact of the matrix on the electrochemical double layer. This observation is supported by the fact that many experts in electrochemical sensors emphasize the importance of a high surface quality for high-performance sensing [23,24]. One way to mitigate these effects is to use labels, which are discussed in the following section.

### D. Labels

Labels serve two purposes. First, they can be an essential part of the sensing mechanism; fluorescence-based sensors would not work without a fluorescent label because many biomarkers of interest do not exhibit autofluorescene. The same is true in electrochemistry, where redox labels are commonly used, as most biomarkers of interest are not redox-active. Without labels, these modalities would not work at all. The well-known Abbott iStat system, for example, uses such electroactive secondary antibody labels [25]. The second reason is to amplify the response and improve the signal/noise ratio.

The downside of the addition of labels is that it increases the complexity of the sample preparation and/or the fluidic sample handling. Therefore, the ultimate dream is to produce “label-free” sensors, yet this needs to be considered in the context of the required performance.

In most diagnostic tests used clinically, labels are introduced by conjugation to a secondary binder molecule, the so-called sandwich assay. As with label-free surface affinity biosensors, sandwich assays rely on a surface-immobilized binder molecule; however, sandwich assays use a two-step process: the target antigen first binds to an immobilized binder molecule, then to a secondary binder, which is conjugated to a label, such as a redox-active molecule or fluorophore. The use of two, sequential affinity binding stages, together with careful washing, can be used to minimize the impact of non-specific binding. Labelled assays thus provide a distinct performance advantage in terms of reduced sensitivity to fouling and thus higher detection specificity.

Often, the label is an enzyme, which also provides an enhanced signal via amplification; such tests are usually referred to as enzyme-linked immunosorbent assays (ELISA), and these are the gold-standard clinical assays at present. ELISAs beautifully combine the advantages of high specificity from the use of two binding events with enzymatic signal amplification. Traditional ELISAs with optical readout typically achieve limits of detection in the low pg/ml range. In electrochemistry, the corresponding example of an enzyme-labelled sensor is the Abbot iStat, which has a reported limit of detection for the cardiac biomarker BNP in human serum of 5 pg/ml [26]. A related example is provided by Arya *et al.* [27] who used an enzyme-linked electrochemical assay in undiluted serum to detect TNF-alpha down to 100 pg/ml levels.

An advantage of electrochemical sensors, due to the fact that the potential has a similar penetration depth as the molecular layer [Figs. 1(c) and 1(d)], is that a change in current can also be measured when a label attached to a linker undergoes conformational change. An example is provided by [13], who used the redox active molecule methylene blue attached to a DNA aptamer that undergoes a conformational change upon binding to its target, changing the position of the methylene blue relative to the electrode, resulting in a change of the rate of electron transfer and thus the measured current.

Photonic labelled methods can achieve even higher performance. A non-enzymatic labelled approach that has demonstrated extremely high performance uses the strategy of attaching nanoparticles to the secondary antibody. These nanoparticles can also be understood as “amplifiers”, because the photonic response to the label is much stronger than to the antigen alone. This method has demonstrated the detection of both proteins and microRNA at fg/ml levels [28] and has recently been commercialized by Iris Kinetics.

#### 1. Chemiluminescent Labels

In addition to fluorescent and enzymatic labels, photonic biosensors that employ chemiluminescent labels are available. Here, limits of detection in the fg/ml range have been reported, for example, 100 fg/ml for troponin in human serum [29]. This raises the question as to why chemiluminescence labels perform so well. We suggest an explanation based on the energy transfer/Faradaic principle outlined in Fig. 1. An electrochemical Faradaic sensor requires the application of a voltage, which will inevitably generate a background current. The equivalent photonic sensor requires a pump laser to excite the fluorescence. While high-quality filters are used to suppress the pump at the emission wavelength, these are not perfect, so some background excitation signal always reaches the detector. In contrast, in chemiluminescence, there is no need for excitation; the chemical reaction alone leads to the emission of photons, so the sample is fundamentally much “darker” and exhibits no background signal due to a pump. Measuring low signals is always easier when there is no background, which chemiluminescence achieves as a photonic technique.

#### 2. Enzyme Switch

The downside of sandwich and ELISA assays is the stringent washing that is required to achieve the high performance, which in turn requires more complex sample handling procedures. An interesting wash-free alternative, which maintains the two-binding event stringency to preserve specificity as well as the enzymatic amplification, is chimeric protein switch biosensors [30]. These sensors are inspired by the glucose sensor that exploits the high specificity of an enzyme to the analyte while also providing signal amplification. Since no such enzymes exist for the vast majority of clinically relevant biomarkers, split enzyme assays can provide an alternative. Here, an enzyme is split into two individually inactive subunits, and each subunit is attached to a recognition element that binds to a unique site on the antigen. Upon the binding of both recognition elements, the two halves of the enzyme are brought in close proximity; they reconstitute and render the enzyme active again [31]. The same idea can be realized with an inhibitor to the enzyme as the second unit, resulting in an enzyme switch for biosensing [30,32,33]. Enzyme switch assays do not rely on washing steps and can be carried out in a single step, and therefore meet the rapid point of care requirement. Applications include the rapid identification of infection markers, as well as therapeutic drug monitoring for monoclonal antibody therapies.

#### 3. Fluorescence and Multicolor Operation

In addition to increasing performance, fluorescent labels also significantly increase functionality, because of the large number of available fluorophores, which directly translates into detection channels. While there are similarly a number of different redox probes that could be used for electrochemical detection, the peak width is broad and the peak maxima highly dependent on local environment, which makes multiplexed measurements challenging. Having fluorophores available that emit or absorb at many different wavelengths is widely used in fluorescence imaging to perform multiparameter analysis, which is also used in multiplexed ELISA assays. An interesting variant is to use the multicolor capability of plasmonic nanoparticles to represent different concentrations via different colors [34]. Clearly, color is a useful parameter for increasing functionality, and it is a major opportunity for photonics.

### E. Fingerprinting

Another unique opportunity for photonics is the ability to conduct “fingerprinting” vibrational spectroscopy, by exploiting the Raman effect or by directly probing vibrational states in the mid-IR. As the name suggests, vibrational spectroscopy probes the vibrational modes of molecules, which allows the identification of specific bonds according to their vibrational fingerprint. As with the multicolor capability of fluorescence spectroscopy, there is no immediate equivalent in electrochemistry. Fingerprinting does not require the surface immobilization of binder molecules, so does not strictly fall into the category of “surface affinity” biosensing; nevertheless, we would like to highlight fingerprinting as an interesting and uniquely photonic opportunity. Importantly, fingerprinting can be used to identify drug molecules, which, owing to their small mass, are very difficult to detect using affinity biosensors, giving rise to the field of therapeutic drug monitoring [35].

**Fig. 6.**
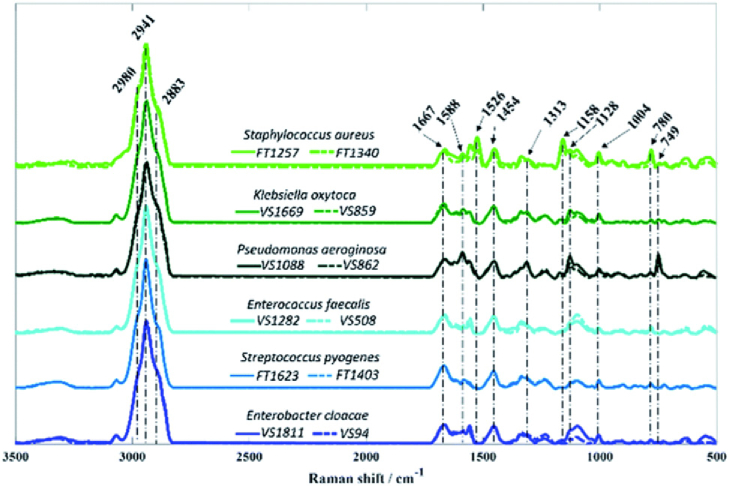
Raman spectra of six bacteria associated with sepsis, (reprinted from [40], following the terms of Creative Commons 3.0 license). There are subtle differences in the $1700\;{cm}^{-1}$ to $500\;{cm}^{-1}$ range, which can be used to identify the respective microbes if appropriate algorithms are applied.

While fingerprinting is very attractive, especially in terms of the range of clinically relevant targets that can be detected (e.g., drug molecules) and novel sensor architectures, there are major interesting scientific and technical barriers that need to be overcome. First, the Raman effect has a very low cross-section, of order $10^{-6}$ of the physical cross-section of the molecule, leading to very weak signals. Using nanoparticles to enhance the Raman effect is a common strategy, resulting in surface-enhanced Raman scattering (SERS), or surface-enhanced resonant Raman scattering (SERRS), the latter resonantly enhancing both the pump and the emission wavelengths. SERS/SERRS are very active areas of research, and good reviews are available, e.g., [36]. We also note the recent emergence of waveguide-based Raman spectroscopy [37,38], which benefits from the longer interaction length offered by the waveguide approach. Nevertheless, the limit of detection of Raman techniques for clinically relevant molecules tends to be much higher than for affinity-based techniques, i.e., it is typically in the µg/ml or high ng/ml range [36]. Raman also needs high-quality lasers and filters, which are expensive. Conversely, mid-IR techniques exploit the much higher cross-sections of molecules in their fundamental vibrational state, but here, photonic technology is far inferior than in the visible and short-wave infrared. For example, the detectivity $D^{*}$ of a silicon photodiode can easily reach $10^{14}$ Jones, while mid-IR detectors can reach $10^{9}$ Jones at best, five orders of magnitude lower. Moreover, water absorption presents a major obstacle, which motivates the use of dried serum spots [39]. Even then, the protein background can easily screen the molecule of interest, leading to limits of detection in the mg/ml or high µg/ml range. On the other hand, mid-IR spectra tend to be very rich and disease-specific correlations have been shown. So rather than focusing on a specific molecule or a single spectroscopic line, the real advantage of mid-IR spectroscopy is to identify disease-specific anomalies, e.g., for the identification of cancer [39]. Another example is bacterial typing, i.e., the ability to identify bacteria; Fig. 6 shows an example.

This example highlights the major difficulty with this approach, namely, that the various spectra look very similar and it is difficult to be specific. Traditionally, principal component analysis (PCA) has been used, which is an elegant method that looks for the largest deviation between datasets. It is increasingly being recognized [41] that machine learning techniques will have a major role to play as long as large datasets for training and testing are available. We suggest that these techniques, if properly applied, will help to improve the specificity of vibrational fingerprinting spectroscopy.

### F. Combination of Modalities

Interesting opportunities emerge at the interfaces between analytical techniques. For example, combinations of plasmonic and electrochemical sensors have successfully exploited the optical enhancement of electrochemical effects to improve performance and amplification effects [42]. What is even more interesting is when the combination of modalities offers a new functionality. In this context, Juan-Colas *et al.* [43] demonstrated the ability to perform electrically controlled functionalization of a photonic sensor array. Here, single-stranded DNA probes were electrochemically grafted onto a photonic sensor surface, thereby controlling the local attachment of binder molecules electrically, while allowing optical detection.

### G. Manufacturing, Scaling-Up, and Stability

Electrochemical sensors have a major technological advantage; electrical contacts and interconnects can be printed easily, at very low cost and the readout electronics can be produced very cheaply, because both have been developed by the microelectronics industry. Electrochemistry therefore offers a true low-cost opportunity. Nevertheless, photonics is catching up with the development of foundry-based silicon photonics, which has now reached a high level of maturity [44]. Photonics can also leverage developments in other markets, for example, low-cost light sources such as diode lasers and LEDs are now readily available at < 1US $ /unit, or high-performance CMOS cameras that have been perfected for the smartphone industry and that also only cost a few US $ per unit.

**Fig. 7.**
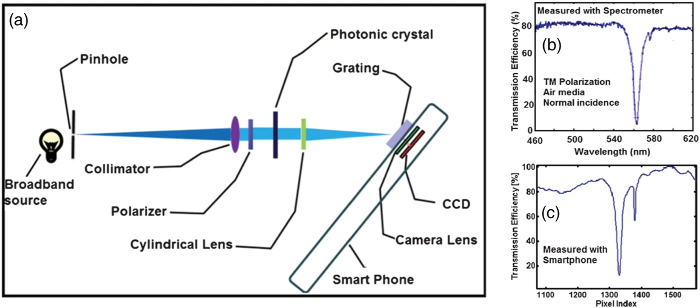
Smartphone-based biosensor, using the camera of a smartphone as the readout element. (a) Schematic; (b), (c) comparison of the readout from an external grating and the smartphone camera (adapted with permission from [45], copyright 2013, Royal Society of Chemistry).

Moreover, photonic modalities can be adopted to directly exploit the capabilities of smartphone technology, given that every smartphone comprises an LED source and one or more cameras. An early example was presented by Cunningham in 2013 [45] where the authors dispersed the information from a resonant grating onto a smartphone camera sensor. A schematic of the arrangement is shown in Fig. 7(a), together with a comparison of the same grating measured with a spectrometer [Fig. 7(b)] and the smartphone [Fig. 7(c)]. The close resemblance of the two spectra highlights the validity of the approach.

While this approach is clearly elegant, and it is easy to imagine how the smartphone LED can be integrated into the optical path, the plurality of available smartphone models and their constantly changing form-factor would present a major challenge to commercializing this approach, as the cradle attaching the sensor to the phone would need to be adapted to every new model on the market.

Therefore, we see the main utility of smartphone technology in providing an easily accessible computing platform and user interface, which minimizes the compute-power required inside the sensing device. This approach is now widely being adopted by the industry.

Finally, let us consider the chemical stability of sensors. Photonic devices, especially dielectric ones, can be made of inert materials or can be suitably coated for long-term stability [46]. In contrast, electrochemical sensors, especially those that exploit Faradaic processes, require a current flow, which tends to cause corrosion of the electrode. More generally, both Faradaic and non-Faradaic modalities measure the potential of the working electrode relative to a reference electrode with a known, fixed, and reproducible potential. The most commonly used reference electrode is the Ag/AgCl electrode. In a laboratory Ag/AgCl reference electrode, the saturated electrolyte is typically contained within a glass tube that enables ionic transport but inhibits leakage of the electrolyte solution, but this is less suitable for portable, low-cost devices. Therefore, solid state reference electrodes have now also been developed as an alternative. While significantly more robust and cheaper than traditional glass-based reference electrodes, these advantages come at the expense of electrochemical performance in terms of potential stability, lifetime, drift rate, and shelf-life. However, for most clinical applications where detection occurs over short timescales (tens of minutes), these low-cost, screen-printed electrodes have proven to be highly effective. Therefore, while the stability of a screen-printed reference electrode may be an issue for long-term measurements, electrochemical sensors can be considered stable in most practical cases.

## 4. TRANSLATION

Finally, we would like to address the question of why, despite significant financial support and associated technological progress, there are so few diagnostic devices that have been successfully commercialized and translated into clinical use. We suggest a few possible explanations.

### A. Performance

A 2022 report [47] highlights that despite many commercial and clinical opportunities, most novel diagnostic tests do not offer the same performance that is already achievable with conventional, laboratory-based tests. It is important to note that in this context, diagnostic performance relates to the sensitivity, specificity, and repeatability of a diagnostic technology in real, clinical matrices rather than in artificial samples such as spiked buffer. It is clearly important and appropriate to initially develop and evaluate a new diagnostic approach using controlled samples and environments. Nevertheless, consideration of the technological challenges facing the integration of a novel biosensor with clinically relevant matrices not only encourages objective assessment of the potential impact of an innovation but also helps inform future research questions.

### B. Clinical Value and Importance

It is interesting to note that commercially successful biosensor technologies all focus on healthcare challenges in which the diagnostic test can be readily integrated into a clinical pathway and inform an actionable outcome. For example, the glucose test enables monitoring of a pre-diagnosed condition and the results inform management of diabetes. Similarly, the lateral flow pregnancy test is used to provide an initial assessment that is subsequently confirmed by clinical evaluation. In contrast, a test designed to provide diagnosis of a life changing condition such as cancer, or the replacement of an existing assessment of life-critical conditions such as the diagnosis of sepsis, faces much higher hurdles in terms of performance and acceptance. It is therefore important to develop and evaluate a new technology in partnership with healthcare professionals who can inform the clinical need and utility.

### C. Social, Political, and Economic Context

The context into which an intervention is used is as critical as the technology itself in determining the outcomes and acceptance. This was revealed starkly during the Covid-19 pandemic, where, without social and political pressure, it is unlikely that the relatively poorly performing lateral flow tests developed early in the pandemic would have been approved for wide scale use. Yet, it is widely accepted that these tests contributed to the ability to monitor and control the spread of infection and to the public’s perception of the pandemic. In the future, we may see similar policy pressures emerging due to the burden of the ageing society or the escalation of antimicrobial resistance, creating new opportunities for the translation of biosensor technology. Additionally, we see opportunities for tests for neurodegenerative disease or cancer that can be deployed at the point of need, driven by policy aimed at addressing the prevalence of these diseases in society.

### D. User-Friendliness

Diagnostic tests designed for use at point of care need to be easy to use (which includes sample extraction, processing and delivery to the sensor), simple to interpret, robust, and fail-safe so to minimize user-induced error; the ubiquitous lateral flow pregnancy-test is a prominent example of such a diagnostic technology designed for home use. Would every one of the high-performance diagnostic technologies that are published in high-profile journals meet this requirement?

### E. Fragmented Application-Space

It is often said that sensors are the next big market opportunity for the microelectronics or silicon photonics industry. Such a statement sounds attractive, and given the growing needs of an ageing society, realistic. There is a catch, however; the existing markets served by silicon technology only deal with “hard”, inorganic materials. As we have highlighted on many occasions throughout this paper, the “soft”, organic biointerface required for the sensor to interact with the matrix adds complexity and application-specificity; one can simply not use the same sensor and biointerface for detecting sepsis and heart attack. Accordingly, the market is fragmented and it is more difficult to benefit from economies of scale. Manufacturers respond to this challenge by developing platforms that can address different diseases via a range of bespoke cartridges that can be analyzed by the same instrument such as the Abbott iStat or the Roche Cobas. An alternative solution would be to develop spectroscopic approaches such as Raman or mid-IR that are much more molecule-agnostic, even though they do not (yet) reach the required limits of detection for many diseases. Moreover, sensors for healthcare applications require formal approval from health agencies such as the FDA in the US, which adds costs and incurs delays. More importantly, the approval is informed by both disease and the technology, which adds further overhead. For this reason, many companies aim for the scientific research market, with Biacore and more recently, IrisKinetics prime examples, which, despite being a smaller market, offer higher margins and lower overheads.

### F. Summary - Translation

We note that similar comments have been made before, yet we believe that they are worth repeating. For example, Zucolotto [24] highlights “the difficulties of manufacturing robust and reliable devices, with good specificity, sensitivity, and above all, reproducibility on a large scale. These challenges apply for all application sectors, including medical applications which, despite having the largest number of commercially available devices, still suffers from a lack of products that can meet the needs of point-of-care applications (portable, low-cost, fast response, disposable).”

Correspondingly, the research community should take a more holistic view and provide a more objective assessment of the clinical value of a new technology if it aims to translate this technology, rather than making over-inflated claims; we note this applies equally to authors, reviewers, and editors. Journals like to report “the next best thing”, but they also have a responsibility to put scientific results into context. Clearly, curiosity-driven, fundamental research into novel biosensor architectures or phenomena is still highly valuable, but this research should also consider reality; if advocating a “near-patient” or “point of care” test, researchers should report analytical performance using real sample matrices, such as blood, serum, or urine. Researchers should also consider the context of their experiment; if a biosensor requires highly tuned and expensive laboratory equipment, or it relies on unconventional or non-scalable fabrication approaches, translation of the technology will face high barriers. We suggest that the potential to translate a given technology should be considered at an early stage.

Finally, it is common practice in many areas of the physical sciences to report results from a few or even a single experiment based on the frequently correct assumption that physical systems are well-behaved and reproducible. This assumption often does not hold for biological and clinical applications. Biosensor research should therefore be informed by best practice in experimental biological and clinical science and report statistically relevant results from multiple experiments and associated control assays.

If the community were to adopt these practices, research output would be even more valuable and, as a result, it would collect more citations. It would promote much needed interdisciplinary collaboration across the chemical, biological, and clinical sciences and increase the commercialization and translation of innovations into the market. In the context of this paper, we note that photonics researchers have typically trained as physicists or electrical engineers, whereas electrochemical sensors span engineering, biochemistry, and chemical engineering. As a result, electrochemical sensor research tends to take a broader view and appreciates interdisciplinary interactions and challenges, an attitude that the photonic sensor community could learn from.

## 5. DISCUSSION AND CONCLUSION

Our analysis has established that both photonic and electrochemical modalities have many issues in common and that the differences are smaller than sometimes claimed. Furthermore, we believe that the classification put forward in Fig. 1 is highly instructive, as it highlights that many properties of the two modalities can be explained via the overlap of the (optical, electric) field with the bound molecular layer, including sensitivity, response to conformational changes, susceptibility to fouling, and surface quality. In particular, the overlap argument explains why electrochemical sensors may be more concerned with surface fouling than photonic ones and why matrix effects impact more on the quantification of electrochemical sensors than on photonic ones.

**Table 1.** Comparison of the Different Modalities Discussed in this Paper, for Detection in Clinical Matrices, Mainly Blood/Serum^*a*^

Method	Type	Limit of Detection	Example
Electrochemical glucose sensor	Elec	µg/ml	[11]
Raman spectroscopy	Phot	µg/ml	[36]
Surface plasmon resonance, e.g., Biacore	Phot	ng/ml	[48]
Label-free photonic blood	Phot	ng/ml	[14]
Label-free photonic urine	Phot	pg/ml	[22]
Faradaic label (e.g., Abbott iStat)	Elec	pg/ml	[27]
Labelled photonic (e.g., ELISA)	Phot	pg/ml	[49]
Chemoluminescence	Phot	fg/ml	[29]
Non-Faradaic EIS	Elec	fg/ml	[10]
Nanoparticle-labelled interferometry	Phot	fg/ml	[28,50]

^*a*^ Please note that the quoted values are approximate and that the quoted references are meant to provide representative examples. Also note that the values reported in laboratory buffers, especially for label-free photonic approaches, tend to be much better, but are deliberately not reported here.

The use of labels is widespread, in fact it is essential for the detection of many target molecules by electrochemistry, because most targets are not electrochemically active. More importantly, labels can be used to increase the sensor specificity and can improve limits of detection. Labels add complexity to the sample preparation, however, which is an issue for the simplicity requirement of the point of care application. Therefore, the vision of developing a label-free, high-performance sensor is still very much alive, especially if it addresses a suitable disease, can be conducted in a clinical matrix, and offers reliable performance that informs a clinically actionable outcome. In addition, we note that some of the most successful point of care sensors, i.e., the glucose sensor and the lateral flow pregnancy test, both detect relatively high levels of target molecules; low-concentration testing is still very much confined to laboratory instruments. This state of affairs offers a clear opportunity for the research community.

For example, the photonics community can learn from the highly successful ELISA assay and aim to miniaturize and simplify it without compromising performance; such a “handheld ELISA” would be very attractive. It requires interdisciplinary expertise, combining photonics with microfluidics and surface chemistry. Elegant solutions are required that provide high performance at low cost. We summarize our comparison of the different modalities in Table 1.

Regarding interdisciplinarity and clinical relevance, photonics researchers can clearly learn from electrochemistry. When conducting our analysis, we noted that electrochemistry papers tend to approach the technological and clinical challenges more holistically; they often measure in clinical matrices, and perform statistically relevant numbers of repeat measurements with appropriately designed control assays. In contrast, there still are many photonics papers that only focus on a single parameter, such as sensitivity or Q-factor. Clearly, there is a lot of value in studying new modalities, but researchers should consider whether there is a realistic pathway towards a clinical application before they make related claims. The quest for the ultimate sensitivity or figure of merit for a photonic biosensor is misguided for the purpose of clinical applications if it fails to consider the limitations of the binder molecule or the impact of fouling, or if it requires highly stabilized laboratory equipment to operate.

Judging by market penetration and number of published papers, electrochemistry is the clear commercial winner, yet we suggest that photonics may overtake electrochemistry in due course. This judgement is informed by the recognition that some of the most highly performing modalities, such as ELISA and chemiluminescence, but also the use of gold nanoparticle labels, are all photonic. Moreover, the multicolor operation and the Raman/mid-IR fingerprinting capabilities are unique in photonics, offering a richer parameter space to explore. We believe that the application of machine learning techniques in this space presents a major opportunity that will provide more reliable disease-specific data.

We trust that our analysis and the insights we offer will inform future research and allow the field to realize its full potential, both scientifically and in terms of translation, commercialization, and clinical impact.

## Data Availability

The data that support the findings of this study are available from the authors upon reasonable request.
